# Molecular epidemiology of extended-spectrum beta-lactamase-producing-*Klebsiella* species in East Tennessee dairy cattle farms

**DOI:** 10.3389/fmicb.2024.1439363

**Published:** 2024-09-24

**Authors:** Benti D. Gelalcha, Ruwaa I. Mohamed, Aga Edema Gelgie, Oudessa Kerro Dego

**Affiliations:** ^1^Department of Animal Science, The University of Tennessee, Knoxville, TN, United States; ^2^Department of Genome Science and Technology, The University of Tennessee, Knoxville, TN, United States

**Keywords:** *Klebsiella pneumoniae*, beta-lactamase genes, dairy farm, genetic diversity, plasmids, transmission, whole genome sequence

## Abstract

**Introduction:**

The rising prevalence of Extended-Spectrum Beta-Lactamase (ESBL)-producing *Klebsiella* species (spp.) poses a significant threat to human and animal health and environmental safety. To address this pressing issue, a comprehensive study was undertaken to elucidate the burden and dissemination mechanisms of ESBL-*Klebsiella* spp. in dairy cattle farms.

**Methods:**

Fifty-seven *Klebsiella* species were isolated on CHROMagar™ ESBL plates and confirmed with MADLI-TOF MS and whole genome sequenced from 14 dairy farms.

**Results and discussion:**

Six families of beta-lactamase (*bla*) (*bla*_CTX−M_, *bla*_SHV_, *bla*_TEM_, *bla*_OXY_, *bla*_OXA,_ and *bla*_SED_) were detected in ESBL-*Klebsiella* spp. genomes. Most (73%) of isolates had the first three types of beta-lactamase genes, with *bla*_SHV_ being the most frequent, followed by *bla*_CTX−M_. Most (93%) isolates harbored two or more bla genes. The isolates were genotypically MDR, with 26 distinct types of antibiotic resistance genes (ARGs) and point mutations in *gyrA*, *gyrB*, and *parC* genes. The genomes also harbored 22 different plasmid replicon types, including three novel IncFII. The IncFII and Col440I plasmids were the most frequent and were associated with *bla*_CTXM−27_ and *qnrB19* genes, respectively. Eighteen distinct sequence types (STs), including eight isolates with novel STs of *K. pneumoniae*, were detected. The most frequently occurring STs were ST353 (*n* = 8), ST469 (*n* = 6), and the novel ST7501 (*n* = 6). Clusters of ESBL-*Klebsiella* strains with identical STs, plasmids, and ARGs were detected in multiple farms, suggesting possible clonal expansion. The same ESBL variant was linked to identical plasmids in different *Klebsiella* STs in some farms, suggesting horizontal spread of the resistance gene. The high burden and dual spread mechanism of ESBL genes in *Klebsiella* species, combined with the emergence of novel sequence types, could swiftly increase the prevalence of ESBL-*Klebsiella* spp., posing significant risks to human, animal, and environmental health. Immediate action is needed to implement rigorous surveillance and control measures to mitigate this risk.

## 1 Introduction

*Klebsiella* species belongs to the genus *Klebsiella* in the family of *Enterobacteriaceae*, and the bacteria naturally reside in the digestive tracts of healthy animals and humans (Martin and Bachman, 2018). *Klebsiella* spp. is identified as one of the most concerning bacteria involved in carrying and disseminating genes mediating resistance to the highest priority and critically important classes of antibiotics (HPCIA), such as third-generation cephalosporins (3GCs) (Boucher et al., 2009; Navon-Venezia et al., 2017). Studies conducted in human health settings documented that *Klebsiella* spp. is one of the most effective bacteria in disseminating extended-spectrum beta-lactamase (ESBL) genes, which confer resistance to 3GC. The bacteria transfer ESBL genes vertically to their subsequent generation and horizontally by acting as donors via mobile genetic elements (MGEs) (Woodford and Livermore, 2011; Wyres and Holt, 2018).

*Klebsiella* spp. is recognized as one of the most frequent causes of community and hospital-associated infections caused by extended-spectrum beta-lactamase (ESBL)-producing bacteria in Europe (Navon-Venezia et al., 2017) and the US (CDC, 2019). The US Centers for Disease Control and Prevention (CDC) recently reported a 50% increase in ESBL-producing *Enterobacteriaceae* infections over six years from 2012 to 2017 (CDC, 2019). Surprisingly, nearly half of these cases were community-associated and had no prior history of healthcare exposure. This rapid increase has led the CDC to designate ESBL-producing *Enterobacteriaceae*, including *Klebsiella* spp., as a “serious threat” to public health (CDC, 2019). However, as almost all previous studies on ESBL-producing *Klebsiella* spp. were focused on humans (Tadesse et al., 2012; Davis and Price, 2016; Martin and Bachman, 2018), the cause for the rapid increase in ESBL-producing *Enterobacteriaceae* infections in community settings is not yet understood.

As a common colonizer of dairy cattle’s gastrointestinal tract (GIT) and one of the causative agents of coliform mastitis, *Klebsiella* spp. is frequently exposed to beta-lactam antibiotics, especially to 3GCs, which is often used for prophylactic and therapeutic purposes in dairy cattle. Previous studies showed that the prevalence of ceftiofur, a veterinary 3GC-resistant *Enterobacteriaceae*, is significantly higher in dairy cattle than in humans and other food animals, such as pigs and chickens (Tadesse et al., 2012). A recent molecular study indicated a similarity between *K. pneumoniae* isolates from animals and humans, suggesting inter-species transmission of the bacteria (Davis and Price, 2016).

The widespread presence of *Klebsiella* spp. in the GIT of dairy cattle, combined with the frequent use of ceftiofur, a 3GC, in dairy farms, may render dairy cattle a potential reservoir for ESBL-producing *Klebsiella* species (Gelalcha et al., 2022). However, this bacterium has been largely overlooked and understudied in dairy cattle farms. As a result, information on the burden of ESBL-*Klebsiella* spp. on dairy farms and the possible mechanisms of its transmission within and between farms is unknown.

Without a detailed understanding of the burden and mechanisms of the spread of these bacteria within and between farms, it is challenging to effectively control its transmission from dairy farms to humans through different routes. Thus, we hypothesized that dairy cattle may serve as a potential reservoir for ESBL-producing *Klebsiella* spp. and ESBL genes that spread vertically and horizontally in the dairy production system. Therefore, this study aimed to identify ESBL genes and other co-carried resistance genes in *Klebsiella* species and understand how these resistance genes spread through clonal expansion or horizontal transfer.

## 2 Materials and methods

### 2.1 Sample collection and laboratory analyses

We conducted a cross-sectional study in 14 East Tennessee dairy farms. We obtained isolates from various sources, including rectal fecal samples using sterile rectal gloves from dairy cows (*n* = 424) and calves (*n* = 84) and farm environmental samples of manure (*n* = 30), feed (*n* = 15), and water (*n* = 19). Feed and water samples were pooled from multiple feeding and watering troughs on the farm and placed in sterile containers. Manure samples were pooled from different farm pens and placed in sterile collection tubes. All samples were transported to the laboratory in coolers to maintain appropriate temperature and prevent bacterial growth. The samples were processed and plated directly on CHROMagar™ ESBL (DRG International, Inc., Springfield, NJ, USA) as described in Gelalcha et al. (2023a). Presumptive ESBL-*Klebsiella* spp. were confirmed using matrix-assisted laser desorption/ionization-time of flight (MALDI-TOF) as described in our previous publications (Gelalcha et al., 2023a,b). Chromosomal DNA was extracted from all MALDI-TOF-confirmed ESBL-*Klebsiella* spp. using Qiagen MagAttract HMW DNA kit (Qiagen, Germantown, MD, USA) as described in Gelalcha et al. (2023b). The genomic DNA libraries were prepared using the Nextera XT DNA Library prep kit from Illumina Corporation (Illumina Inc., San Diego, CA, USA), following the manufacturer’s instructions. Whole genome sequencing (WGS) was conducted on *Klebsiella* isolates (*n* = 56) using Illumina NovaSeq PE250 at the University of Tennessee Genomics Core (Knoxville, TN, USA). This study was approved by the University of Tennessee’s Institutional Animal Care and Use Committee (IACUC) Registration Number: 2782-0720.

### 2.2 Statistical data analyses

The collected raw data was entered into Microsoft Excel for Windows 10 (Microsoft Corp., Redmond, WA, USA) before being transferred to SPSS (SPSS Statistics for Windows, Version 27.0, IBM Corp, Armonk, NY, USA) for statistical analysis. Descriptive (frequency) statistics were used to summarize the collected data.

### 2.3 Whole genome sequence data processing and analyses

The whole genome sequence data analysis involved demultiplexing of the sequence data, followed by quality assessment using FastQC v0.11.9 (Babraham Bioinformatics, 2024) and multiQC v1.14 (Ewels et al., 2016). Trimmomatic v0.39 was used to remove adapters, filter, and trim raw reads that had low-quality (Bolger et al., 2014). Preprocessed reads of each isolate were then subjected to *de novo* assembly using the default parameters of the SPAdes genome assembler v3.15.5 (Bankevich et al., 2012). BBMap (version 39.06) was used to filter contigs shorter than 500 bp.^1^ QUAST (v5.2.0) was used to generate basic genome assembly statistics (Mikheenko et al., 2018). For downstream analysis, PlasmidSPAdes modes were used to assemble only the plasmids. Several web-based online bioinformatics tools were used to analyze the genetic characteristics of bacterial isolates. To confirm bacterial spp. KmerFinder 3.2^2^ (Hasman et al., 2014; Larsen et al., 2014; Clausen et al., 2018) and https://pubmlst.org/bigsdb?db=pubmlst_rmlst_seqdef_kiosk were used. Antibiotic resistance genes (ARG) and mutations conferring resistance were detected using^3^ (Zankari et al., 2017; Bortolaia et al., 2020) and CARD^4^ (Alcock et al., 2020). Plasmids associated with ARGs were identified using plasmid finder^5^ (Camacho et al., 2009; Carattoli et al., 2014), and their sequence types were characterized using pMLST 2.0^6^. Mobile genetic elements (including plasmids) associated with ARGs were identified using MobileElementFinder^7^ (Camacho et al., 2009; Zankari et al., 2012; Joensen et al., 2014). The ESBL-*Klebsiella* spp. sequence type (ST) was determined based on seven housekeeping genes (*gapA*, *infB*, *mdh*, *pgi*, *phoE*, *rpoB*, and *tonB*) as described in Diancourt et al. (2005). The ST of the *Klebsiella* spp. were assigned using tools at the Center for Genomic epidemiology,^8^ and the *Klebsiella* Pasteur MLST sequence database.^9^ The combination of the best matching allele was selected to determine the sequence type of each *Klebsiella* spp.

Phylogenetic analyses of the ESBL-*Klebsiella* species isolates were automated using GToTree (version 1.8.6) (Lee, 2019). The GToTree default parameters were used to build the tree based on nucleotide sequence alignment using “Bacteria” genes. GCF 000240185.1 *K. pneumoniae* HS11286 was used as the reference genome. Interactive Tree of Life (iTOL version 6) was used to visualize the tree interactively online.

The *Klebsiella* species genomes have been submitted to the Pasteur Institute bacterial isolate Genome Sequence Database BIGSdb_20240715165555_3499682_34488.

## 3 Results

### 3.1 Microbiological and whole genome sequence (WGS) result of *Klebsiella* species

Using MALDI-TOF MS, we identified 57 *Klebsiella* spp. consisting of four distinct species from 14 farms (Supplementary Table 1). These include *K. pneumoniae* (*n* = 52), *K. variicola* (2 isolates), *K. oxytoca* (2), and *K. aerogenes* (1).

Of the 57 *Klebsiella* isolates identified, 56 were subjected to WGS. The MALDI-TOF MS test misidentified two *K. pneumoniae* isolates as *K. variicola*, later confirmed by WGS analysis. Therefore, the total number of *K. pneumoniae* isolates sequenced was 53. Two isolates initially identified as *K. oxytoca* by MALDI-TOF MS from the same farm were later determined to be *K. michiganensis* through WGS analysis. One *K. michiganensis* genome was contaminated with the *E. coli* genome (43%) and excluded from further downstream WGS analysis. Thus, further genomic analysis was carried out on the remaining 55 *Klebsiella* spp (53 *K. pneumoniae* and one from *K. michiganensis* and *K. aerogenes*). Summary statistics related to *Klebsiella* species genome assembly, such as coverage, number of contigs, N50, and others, were given in Supplementary Figure 1.

### 3.2 Genetic determinants of resistance to beta-lactam antibiotics

All sequenced isolates had one or more kinds of ESBL-encoding genes. This study detected six families of beta-lactamase genes from the ESBL-*Klebsiella* spp. genomes. These include *bla*_CTX−M_, *bla*_SHV_, *bla*_TEM_, *bla*_OXY_, *bla*_OXA,_ and *bla*_SED_. The majority (73%; 40/55) of the isolates harbored three types of beta-lactamase genes, namely *bal*_CTX−M_, *bla*_SHV_, and *bla*_TEM_, in their genomes. Only five isolates carried a single kind of beta-lactamase gene, four of which had *bla*_SHV−2,_ and the remaining one had *bla*_CTX−M_. The remaining ten isolates had two beta-lactamase genes in their genomes. In this manuscript, we will focus only on ESBL variants and inhibitor-resistant beta-lactamase genes in each family.

Interestingly, thirteen isolates of *K. pneumonia* had inhibitor-resistant *bla*_SHV_ genes, either *bla*_SHV−56_ or *bla*_SHV−26_ (Table 1). In addition, inhibitor-resistant *bla*_TEM_ variant beta-lactamases, including *bla*_TEM−33_, *bla*_TEM−34_, *bla*_TEM−35_, *bla*_TEM−36_, and *bla*_TEM−122_, were each detected in five separate isolates.

**TABLE 1:** Beta-lactamase genes, their function, and frequency in ESBL-*Klebsiella* species.

Farm	β -Lactamase gene variant	Functional information	Frequency
A, B, G, L, M	*bla*_CTX−M−1_	Class A ESBL	12
M	*bla*_CTX−M−15_	Class A ESBL	1
M	*bla*_CTX−M−27_	Class A ESBL	37
M	*bla*_CTX−M−32_	Class A ESBL	2
M	*bla*_CTX−M−61_	Class A ESBL	2
M	*bla*_CTX−M−65_	Class A ESBL	5
M	*bla*_CTX−M−138_	Class A ESBL	2
M	*bla*_CTX−M−146_	Class A ESBL	2
M	*bla*_SHV−1_	2b Class A β-lactamase	1
J, M	*bla*_SHV−2_	2be Class A β-lactamase	4
M	*bla*_SHV−11_ *^c^*	2b Class A β-lactamase	8
M	*bla*_SHV−13_ *^c^*	2be Class A β-lactamase	8
M	*bla*_SHV−110_	Class A β-lactamase	1
A B, G, M	*bla*_SHV−85_ *^a^*	2b Class A β-lactamase	5
A, B, G, M	*bla*_SHV−40_ *^a^*	2be Class A β-lactamase	5
A, B, G, M	*bla*_SHV−56_ *^a^*	2br Class A β-lactamase	5
A, B, G, M	*bla*_SHV−79_ *^a^*	2b Class A β-lactamase	6
A, B, G, M	*bla*_SHV−89_ *^a^*	2b Class A β-lactamase	5
M	*bla*_SHV−59_ *^d^*	2b Class A β-lactamase	4
M	*bla*_SHV−70_ *^c^*	2be Class A β-lactamase	8
M	*bla*_SHV−71_	2b Class A β-lactamase	1
M	*bla*_SHV−25_	2b Class A β-lactamase	1
M	*bla*_SHV−26_ *^b^*	2br Class A β-lactamase	7
M	*bla*_SHV−27_	2be Class A β-lactamase	9
M	*bla*_SHV−62_	2b Class A β-lactamase	4
M	*bla*_SHV−78_ *^b^*	2b Class A β-lactamase	7
M	*bla*_SHV−81_	2b Class A β-lactamase	1
M	*bla*_SHV−145_ *^b^*	2b Class A β-lactamase	7
M	*bla*_SHV−172_	Class A β-lactamase	1
M	*bla*_SHV−157_	Class A β-lactamase	1
M	*bla*_SHV−187_	Class A β-lactamase	1
M	*bla*_SHV−164_ *^d^*	2b Class A β-lactamase	4
M	*bla_*SHV−98*_*^b^**	2be*Class A β-lactamase	7
M	*bla*_SHV−199_ *^b^*	Class A β-lactamase	7
M	*bla*_SHV−103_	2b Class A β-lactamase	1
M	*bla*_*SHV−78*_	2b Class A β-lactamase	7
M	*bla_*SHV−179*_*^b^**	Class A β-lactamase	7
M	*bla*_SHV−194_ *^b^*	Class β-lactamase	7
M	*bla*_TEM−1B_ *^e, f^*	2b Class A β-lactamase	40
M, G	*bla*_TEM−1C_ *^f^*	2b Class A β-lactamase	2
M	*bla*_TEM−16_ *^e^*	2be Class A β-lactamase	1
M	*bla*_TEM−29_ *^f^*	2be Class A β-lactamase	1
M	*bla*_TEM−33_ *^e^*	2br Class A β-lactamase	1
M	*bla*_TEM−34_ *^e^*	2br Class A β-lactamase	1
M	*bla_TEM−35_*^e^**	2br Class A β-lactamase	1
M	*bla*_TEM−36_ *^e^*	2br Class A β-lactamase	1
M	*bla*_TEM−55_ *^f^*	2b Class A β-lactamase	1
M	*bla*_TEM−57_ *^f^*	2b Class A β-lactamase	1
M	*bla*_TEM−104_ *^e^*	Class A β-lactamase	1
M	*bla*_TEM−122_ *^e, f^*	2br Class A β-lactamase	2
M	*bla*_TEM−135_ *^f^*	2b Class A β-lactamase	1
M	*bla*_TEM−141_ *^e, f^*	2b Class A β-lactamase	2
M	*bla*_TEM−163_ *^e^*	2be Class A β-lactamase	1
M	*bla*_TEM−164_ *^e^*	2b Class A β-lactamase	1
M	*bla*_TEM−198_ *^e^*	Class A β-lactamase	1
M	*bla*_TEM−206_ *^e^*	Class A β-lactamase	1
M	*bla*_TEM−209_ *^ef^*	Class A β-lactamase	2
M	*bla*_TEM−210_ *^e^*	Class A β-lactamase	1
M	*bla*_TEM−214_ *^e^*	Class A β-lactamase	1
M	*bla*_TEM−216_ *^e^*	Class A β-lactamase	1
M	*bla*_OXA−1_	Class D β-lactamase	1
M	*bla*_OXY−1−7_	Class A ESBL	1
M	*bla*_SED−1_	2b Class A β-lactamase	1

*^a−f^*Beta-lactamase genes with the same superscript co-occur in the same isolates and are usually found on the same contigs. 2b: broad-spectrum; 2be: extended-spectrum; 2br: inhibitor-resistant broad-spectrum. The phenotype or functional information of the gene in the table was adopted from the NCBI (https://www.ncbi.nlm.nih.gov/pathogens/isolates//#AMR_genotypes), beta-lactamase database (http://bldb.eu/BLDB.php?prot=A) and https://www.lahey.org/Studies.

Regarding distribution within isolates, *bla*_SHV_ was the most frequent and was detected in 53 isolates, followed by *bla*_CTX−M_ and *bla*_TEM_, which were detected in the genomes of 51 and 40 isolates, respectively. Furthermore, *bla*_SHV_ exhibited the highest level of diversity with 30 identified variants. However, only 40% (*n* = 12) of *bla*_SHV_ variants were ESBL. In most cases (41 out of 53 isolates), multiple variants of *bla*_*SHV*_ were found clustered together on the same contigs within the genome of an isolate, creating a resistance island.

Of the 51 isolates containing *bla*_CTX−M_, only three had more than one variant of the *bla*_CTX−M_ gene, while 48 had only one variant. However, two *Klebsiella* isolated from the same farm harbored six distinct variants of the *bla*_CTX−M_ genes per genome (Table 1). These variants include *bla*_CTX−M−27_, *bla*_CTX−M−146_, *bla*_CTX−M−32_, *bla*_CTX−M−61_, *bla*_CTX−M−138_, and *bla*_CTX−M−1_. All these genes were located on the same contigs except for *bla*_CTX−M−27_. In this study, *bla*_CTX−M−27_ was the most frequent *bla*_CTX−M_ allele, followed by *bla*_CTX−M−1_ and *bla*_CTX−M−65_ (Table 1). Among the two rare *Klebsiella* spp. reported in this study, *K. michiganensis* had *bla*_CTX−M−1_ and *bla*_OXY−1−7_ genes, while *K. aerogenes* had *bla*_CTX−M−65_.

Among 24 different variants of *bla*_TEM_, the narrow spectrum *blaTEM*_−_*_1B_* was detected in 97.6% (40/41) of *Klebsiella* isolates carrying this gene, making it the most prevalent variant. Only two isolates possessed *bla*_TEM−1c_. The remaining 22 *bla*_TEM_ variants were found in just one *Klebsiella* isolates each. Among the detected *bla*_TEM_, only three of them, bla*_TEM−16_*, *bla_TEM−29_*, and *bla*_TEM−163_, were categorized as ESBL variants. One isolate harbored seventeen *bla*_TEM_ variants, including four inhibitor-resistant beta-lactamases (*bla*_TEM−33_, *bla*_TEM−34_, *bla*_TEM−35_, and *bla*_TEM−36_) and fourteen other variants (indicated with the superscript “e” in Table 1) on a contig. Similarly, other *Klebsiella* isolates from the same farm harbored nine *bla*_TEM_ variants, all co-located on the same contigs (the genes are indicated with the superscript “f” in Table 1).

### 3.3 Detection of horizontally transferable resistance genes in ESBL-*Klebsiella* isolates

Apart from beta-lactamase genes, *Klebsiella* spp. harbored resistance genes against eight different classes of antibiotics in their genomes (Table 2). All 55 *Klebsiella* isolates were genotypically multidrug resistant, carrying resistance genes to as high as seven classes of antibiotics in their genomes. In addition to the beta-lactamase genes, about 26 resistance genes mediating resistance to about eight classes of antibiotics were detected. These include resistance genes against (fluoro)quinolones, macrolides, aminoglycosides, folate pathway inhibitors, tetracycline, chloramphenicol, lincosamides, and phosphonic acids.

**TABLE 2:** Horizontally transferable resistance genes co-harbored with beta-lactamase genes in *Klebsiella* species.

Target antibiotic class	Resistance gene and frequency	Gene description	Resistance mechanism
Beta-lactams	*bla*_CTX−M_ (51)	Class A ESBL	IBH
	*bla*_SHV_ (53)	Class A beta-lactamase	IBH
	*bla*_TEM_ (40)	Class A beta-lactamase	IBH
	*bla*_*OX*_*_*Y*_* (1)	Class A ESBL	IBH
	*bla*_OXA_ (1)	Class D β-lactamase	IBH
	*bla*_*SED*_ (1)	Class A beta-lactamase	IBH
Aminoglycosides	*aph*(6)-Id(3)	Aminoglycoside-6-Phosphotransferase	IBM
	*aph*(3′′)-Ia(1)	Aminoglycoside-3′′ phosphotransferase	IBM
	*aac*(3)-IId(5)	Aminoglycoside3-N-acetyltransferase	IBM
	*aac*(6′)-Ib-cr(1)	Aminoglycoside6′-N-acetyltransferase	IBM
Macrolide	*mph*(A) (9)	Macrolide 2′-phosphotransferase	IBM
(Fluoro)quinolone	*qnrB19* (36)	Fluoroquinolone resistance protein B19	TP
	*qnrB10*(2)	Fluoroquinolone resistance protein B10	TP
	*qnrB5*(2)	Fluoroquinolone resistance protein B5	TP
	*qnrB81*(2)	Fluoroquinolone resistance protein B81	TP
Folate pathway antagonist	*dfrA1*(10)	Dihydrofolate reductase type 1	TM
	*dfrA12*(1)	Dihydrofolate reductase type 12	TM
	*dfrA14*(1)	Dihydrofolate reductase type 14	TM
	*sul1*(3)	Dihydropteroate synthetase type 1	TM
	*sul*2 (2)	Dihydropteroate synthetase type 2	TM
Tetracycline	*tet*(A) (6)	Tetracycline resistance protein A	EM
	*tet*(J) (1)	Tetracycline resistance protein J	EM
Chloramphenicol	*floR*(1)	Florfenicol/ chloramphenicol export protein	EM
	*catB3*(1)	Chloramphenicol acetyltransferase (group B)	IBM
	*cat*(1)	Chloramphenicol acetyltransferase	IBM
Lincomycin	*lnu*(G) (1)	Lincosamide nucleotidyl transferase	IBM
Phosphonic acid	*fosA*(53)	Fosfomycin modifying enzyme	IBM
Multidrug class	*fosA5*(1)	Fosfomycin modifying enzyme type 5	IBM
	*fosA6*(5)	Fosfomycin modifying enzyme type 6	IBM
	*fosA7*(1)	Fosfomycin modifying enzyme type 7	IBM
	*fosA8*(1)	Fosfomycin modifying enzyme type 8	IBM
	*oqxAB*(53)	Efflux transporter	EM

EM, efflux-mediated; IBH, inactivation by hydrolysis; TM, drug target modification; IBM, inactivation by modification; TP, target protection.

Multiple variants of resistance genes against specific classes of antibiotics were detected, such as five variants of each of the fosfomycin and sulfonamide resistance genes and four variants of each of the aminoglycoside and quinolone resistance genes. Among these genes, the most frequently occurring ones were the fosfomycin resistance gene (*fosA*) and multidrug efflux pump encoding genes (*oqxAB*), present in the genome of 53 isolates. The plasmid-mediated fluoroquinolone resistance gene, *qnrB19*, was detected in 36 *Klebsiella* isolates.

Isolates from the same farm exhibited similar resistance gene patterns. For instance, all isolates from Farm B had the same resistance pattern, with respect to the *bla*_CTX−M_ variant type (*bla*_CTX−M−1_), the presence of macrolide resistance gene *mph*(A), and sulfonamide resistance gene (*dfrA1*). Similarly, all isolates from Farm J had *bla*_SHV−2_ genes (no *bla*_CTX−M_ or *dfrA1*). In contrast, identical sequence types between farms showed the same resistance pattern. For example, ST353 detected in three farms (Farm A, B, and J) carried the same gene variants, including *bla*_CTX−M−1_, *mph*(A), *bla*_SHV_, *dfrA1*, *qnrB19*, and *fosA*.

The most frequent chromosomal mutations detected in ESBL-*Klebsiella* genomes were DNA gyrases (*gyrA* and *gyrB*) and topoisomerase IV (*parC*). Multiple mutations that mediate fluoroquinolone resistance, *gyrA*, and *parC* were detected in all 55 *Klebsiella* spp. genomes.

### 3.4 Plasmid replicon types and their association with resistance genes

From the WGS data of 55 ESBL-*Klebsiella* isolates obtained from six farms, 22 different plasmid replicon types were identified. On average, each ESBL-*Klebsiella* isolate harbored five different kinds of replicons, with a minimum of two and a maximum of nine replicon types. Isolates with similar genotypes tend to have similar numbers and replicon types of plasmids. The most frequently detected replicon types in this study were IncF family plasmids (IncFII, IncFIA, and FIB) and Col plasmids such as Col440I, Col(IMGS31), Col(MG828), ColRNAI, Col(pHAD28), ColpVC, Col(BS512), and Col156. Additionally, a limited number of isolates were found to harbor broad-host-range plasmids, including IncH plasmids (such as ncHI2A and IncHI2), IncN, and IncR.

Most (96.4%) of the *Klebsiella* isolates’ genomes harbored the Col440I plasmid. Among the narrow host range, IncF family plasmids, including FII, IncFIA, and FIB replicon types, were highly prevalent, with up to four subtypes of these plasmids present in each isolate. Among the IncF family plasmids, the IncFII plasmid replicon types were the most frequently detected (89%), followed by IncFIB (85%). Notably, all IncFII plasmid replicon types were found to have the same sequence type, ST F2: A^−^: B^−^.

The broad host range plasmid IncN was detected in all nine *K. pneumoniae* with identical STs obtained from three farms (Farms A, B, and G) and *K. michiganensis* strain obtained from Farm L. The IncN plasmid was identified as ST1 in all these isolates and was primarily linked with *bla*_CTXM−1_. On the other hand, the IncH plasmids (IncHI2A and IncHI2) were detected in only three *Klebsiella* isolates with identical genotypes (ST111), which carried *bla*_CTXM−65_. The IncN plasmid was not detected in the remaining 45 isolates obtained from the other farms. The IncR plasmids were identified in only two *K. pneumoniae* isolates in a single farm but were not associated with any of the resistance genes identified in this study. Three Novel IncF RSTs (due to novel FIA allele) were detected in three *Klebsiella* isolates obtained from the same farm. Two of them have the same nearest Sequence type, K4: A^−^: B^−^, and the ST closest to the third novel allele was K7: A^−^: B^−^.

### 3.5 Association between resistance genes and mobile genetic elements

Most isolates (88.5%; 46/52) containing *bla*_CTX−M_ genes were found to have these genes associated with mobile genetic elements (MGEs). In 80% (37/46) of the isolates, *bla*_CTX−M_ was associated with plasmids, while in the remaining 20%, it was associated with insertion sequences (IS) of different types. In 67.4% (31/46) of the isolates, the *bla*_CTX−M−27_ and *bla*_TEM−1B_ genes co-occurred and were associated with epidemic resistance plasmids, IncFII, which had identical plasmid sequence types, STF2: A^−^: B^−^. In six isolates obtained from three different farms, the genes *bla*_CTX−M−1_ and *mph*(A) were both associated with broad host range plasmids of the IncN type, which were subtyped as ST1. The gene *bla*_CTX−M−65_, often detected in ST111, was associated with IncHI2 ST3, IS102, or IS26. Other resistance genes, such as *aph*(6)-Id, *aph*(3′′)-Ib, and *sul*2, were also co-harbored with different variants of *bla*_CTX−M_ on the same MGEs. Similarly, other resistance genes were often associated with MGEs (for example, *qnrB19* was associated withol440I plasmids in 73% of the isolates harboring this resistance gene). Additionally, the *tet(*A) gene was often found to be associated with broad host range plasmids of the IncHI2A type with ST3 (Table 3).

**TABLE 3:** Association between *Klebsiella* species sequence types, resistance genes, and mobile genetic elements.

Farm	ST(Freq.)^a^	Resistance genes^b^	Mobile genetic elements	
			Plasmid replicon type (pMLST)^c^	Insertion sequence
A	ST353 (2)	*bla*_CTX−M−1_, *mph(A)*	IncN (ST1)	−
B	ST353 (1)	*bla*_CTX−M−1_, *mph(A)*	IncN (ST1)	−
G	ST353(2)	*bla*_CTX−M−1_, *mph(A)*	IncN (ST1)	−
M	ST7501 (6), ST 867(3), ST 687(4), ST 469(6), ST 2259(1), ST 239(3), ST 419(1), ST7502(1), ST7503(1), ST 36(1), ST 294(1), unknown ST(2)	*bla*_CTX−M−27_, *bla*_TEM−1B_	IncFII ([F2: A^−^: B^−^])	−
M	ST111(1)	*bla*_CTX−M−65_	IncHI2 (ST3)	
M	ST34(1)	*bla*_CTX−M−27_	IncFII ([F2: A^−^: B^−^])	
M	ST687(1), ST2239(1), ST2703(1), ST7501(1)	*bla*_CTX−M−27_, *bla*_TEM−1B_	−	IS102
M	ST111(1)	*bla*_CTX−M−65_	−	IS102
M	ST469(1)	*bla*_SHV−11_, *bla*_SHV−13_, *bla*_SHV−70_	−	IS102
A	ST353 (1)	*bla*_CTX−M−1_, *mph(A)*	−	IS26
M	ST661(1)	*bla*_CTX−M−27_, *bla*_TEM−1B_	−	IS26
M	ST111(1)	*bla*_CTX−M−65_, *aac*(3)*-Iid*	−	IS26
M	ST2703(1)	*bla*_SHV−199_, *bla*_SHV−179_, *bla*_SHV−45,_ *bla*_SHV−194_, *bla*_SHV−26_, *bla*_SHV−98_, *bla*_SHV−78_	−	IS3
M	ST6250(1)	*bla*_CTX−M−15_, *bla*_TEM−1B_, *aph*(6)*-Id*, *aph(3*′′*)-Ib*, and *sul2*	−	ISEc9
G	ST353 (1)	*bla*_SHV−79_, *bla*_SHV−40_, *bla*_SHV−89_, *bla*_SHV−56_, and *bla*_SHV−85_	−	ICEKp1
All farms	ST353(8), ST322(4), ST7501(3), ST7503(1), ST867(2), ST469(5), ST111(1) ST225(1)	*qnrB19*	Col440I	−
M	ST111(3), ST57(1)	*tet(A)*	IncHI2A(ST3)	−
M	ST2239 (1)	*aph*(6)*-Id*, *aph(3*′′*)-Ib*, *sul2*, *dfrA1*, *floR*, *lnu(G)* and Dihydropteroate synthetase type 2	−	IS91

^a^ST, Sequence type; Freq., number of isolates with the specific sequence type. ^b^Genes co-occurred on the same mobile genetic elements. ^c^pMLST, plasmid multilocus sequence type.

### 3.6 Genetic diversity and possible spread mechanisms of ESBL-*Klebsiella* species in the dairy farms

Eighteen distinct sequence types (STs) of *K. pneumoniae* were identified, including three novel STs (ST7501, ST7502, and ST7503) detected in eight samples. The most frequently occurring STs were ST353 (*n* = 8), ST469 (*n* = 6), the novel ST7501 (*n* = 6), ST687 (*n* = 5), ST322 (*n* = 4), ST111 (*n* = 4), ST2239 (*n* = 4), ST867 (*n* = 3), and ST225 (*n* = 2). The other seven STs were observed only once, including ST7502, ST7503, ST34, ST6250, ST2703, ST36, ST294, ST661, and ST419. The ST *K. aerogenes* was identified as ST57. The ST of *K. michiganensis* was unknown and could not be assigned as the https://bigsdb.pasteur.fr database handles only the *K. pneumoniae* species complex. The remaining five *K. pneumoniae* isolates could not be assigned to specific sequence types because their genome assembly statistics did not meet the criteria set by the database, and alternative databases^8^ provided ambiguous results.

ESBL-producing *Klebsiella* spp. were found to spread both clonally through specific *Klebsiella* spp. STs, and horizontally, via plasmids between genetically unrelated *Klebsiella* spp. Some of the *Klebsiella* STs showed both within-farm and between-farm similarity. ST353, for example, was found in three different farms: three isolates, each from farms A and B, and two isolates from farm G. All these isolates carry the same ESBL variant (*bla*_CTX−M−1_) associated with plasmids with identical sequence types, IncN (ST1). Farms A and B are in the same county, but in different cities, and Farm G is in a different county.

On the other hand, some STs were only detected within a single farm. For instance, all four ST322 isolates were found exclusively in farm J and had the same *bla*SHV-2 variants. One of these STs was isolated from a water sample collected from water troughs.

**FIGURE 1:**
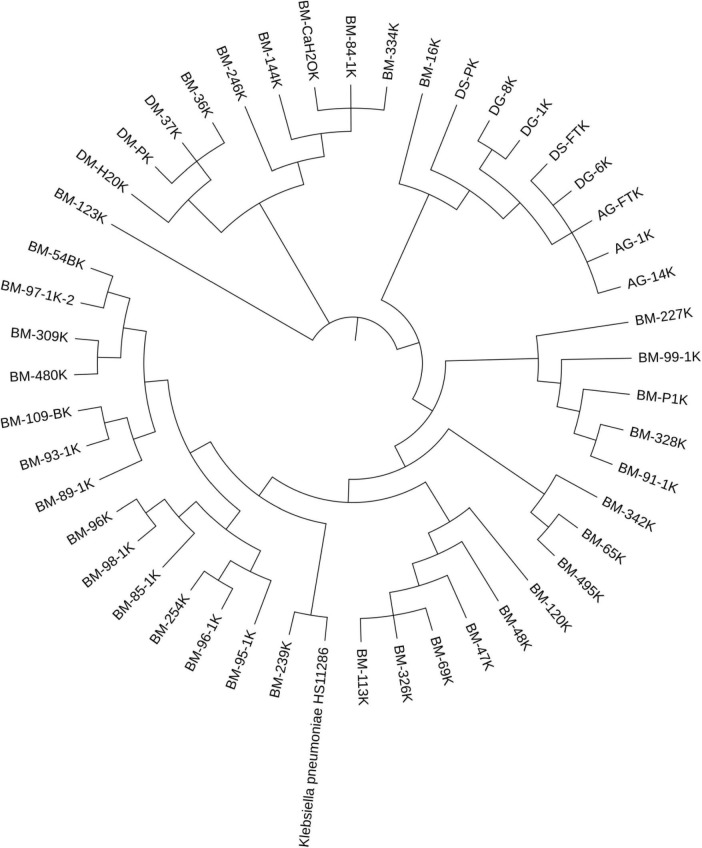
Phylogenetic tree of **ESBL**-***Klebsiella*** *pneumoniae*. Isolates sharing the same first two letters were collected from the same farm. The letters indicate the farm and county, while the numbers represent the individual animal’s ID within the farm (except manure, feed, and water samples). Most of the isolates were obtained from the same farm, and they showed multiple clustering, with isolates in each cluster having identical sequence types.

Farm M had the largest number of sequenced *Klebsiella* isolates (76.4%; 42/55) and displayed the greatest diversity regarding *Klebsiella* STs. It contained 13 distinct sequence types of *K. pneumoniae* and ten unknown STs. The most common STs in this farm include the novel ST7501 (*n* = 6), ST469 (*n* = 6), and ST687 (*n* = 5), followed by ST111 and ST2239, each occurring four times in the farm. Despite the genetic diversity, most isolates in farm M harbored indistinguishable plasmid (e.g., IncFII STF2: A^−^: B^−^) associated with the same variants of ESBL (*bla*_CTX−M−27_). The same ESBL variant was also detected associated with the same plasmid sequence types among diverse STs of *Klebsiella* spp. A similar pattern was also observed for other resistance genes, such as *qnrB19* and *tet*(A) (Table 3). In addition, the phylogenetic tree displayed several clusters of *Klebsiella* isolates within a farm, and some isolates from a different farm were also clustered together (Figure 1).

## 4 Discussion

The detection of at least one ESBL-encoding gene in all sequenced *Klebsiella* spp. generally concurs with the CHROMagar™ ESBL screening result, which shows the high sensitivity and specificity of the chromogenic agar. In this study, detecting six distinct beta-lactamase gene families in *Klebsiella* genomes indicates that these bacteria have an arsenal of genes to inactivate beta-lactam antibiotics and can serve as a reservoir of these genes (Navon-Venezia et al., 2017; Wyres and Holt, 2018). This is particularly problematic given that these resistance genes can transfer from *Klebsiella* spp. to other pathogenic bacterial species that cause life-threatening infections in humans and animals that are difficult to treat with available antibiotics (Wyres and Holt, 2018). This finding agrees with the previous report that indicated *Klebsiella* spp. is a “key source and trafficker” of resistance genes, including ESBL genes (Navon-Venezia et al., 2017; Wyres and Holt, 2018). A similar level of ESBL burden was reported from *Klebsiella* spp. isolated from a food animal in China (Wu et al., 2016).

Among the 30 *bla*_SHV_ variants, The ESBL variants such as *bla*_SHV−27_, _−13_, and _−70_ occurred frequently, and *bla*_SHV−27_ occurred alone, whereas the remaining three co-occurred, suggesting they were relatively widespread among the *Klebsiella* isolates in the farms. We did not find previous reports of *bla*_SHV_ ESBL variants from *Klebsiella* spp. in US dairy farms. However, a study conducted in Oklahoma in 2005 detected *bla*_SHV−1_ from *K. pneumoniae* isolated from cattle farms. It is unclear whether these were beef or dairy farms (Kim et al., 2005). In addition, *bla*_SHV_ variants have been reported in *Klebsiella* spp, isolated from humans in the US (Yigit et al., 2001; Paterson et al., 2003). Some *bla*_SHV_ variants identified in this study, such as *bla*_SHV−11_, have previously been reported in *E. coli* and *K. pneumoniae* isolated from dairy farms in Japan (Ohnishi et al., 2013). In contrast, others, like *bla*_SHV−2_, have been reported in *E. coli* isolated from beef cattle in Canada (Cormier et al., 2016).

The identification of the *bla*_CTX−M_ gene as the second most prevalent ESBL gene in the *Klebsiella* genomes (93%) supports the recent studies that showed this gene family is rapidly disseminating among ESBL-producing *Enterobacteriaceae* (Zhao and Hu, 2013; Bevan et al., 2017; Irrgang et al., 2017; Afema et al., 2018).

Eight *bla*_CTX−M_ gene variants were detected in this study most of which were reported previously from other members of *Enterobacteriaceae*, mainly from *E. coli* isolates from US dairy farms. For instance, *bla*_CTX−M−1_ was reported from *K. pneumoniae* isolates from mastitis milk in Iowa and New York (Yang et al., 2019; Zheng et al., 2022). The *bla*_CTX−M−1_ was also detected from *E. coli* and *Salmonella* isolates retrieved from dairy cattle fecal samples obtained from Ohio, Washington, Texas, and the Southwestern region of the US, suggesting that it is a common *bla*_CTX−M_ variant in dairy farms (Dunne et al., 2000; Cummings et al., 2009; Taylor et al., 2020; Carey et al., 2022). Due to selective pressure, it is possible that *K. pneumoniae* acquired this gene through horizontal transfer from *E. coli* or other sources.

Similarly, *bla*_CTX−M−15_, *bla*_CTX−M−27_, *bla*_CTX−M−32_, and *bla*_CTX−M−65_ were reported from *E. coli* isolated from fecal samples in different parts of the US, including Washington, Texas, and the Southwestern region of the country (Donaldson et al., 2006; Cummings et al., 2009; Afema et al., 2018; Taylor et al., 2020; Carey et al., 2022). The *bla*_CTX−M−61_, *bla*_CTX−M−146_, and *bla*_CTX−M−138_ detected in this study were not previously reported from US dairy farms. In addition, except *bla*_CTX−M−1_, all other bla_CTX−M_ variants were not reported previously from *Klebsiella* isolates from the US dairy farms.

Among the detected *bla*_CTX−M_ genes, *bla*_CTX−M−27_ was the most prevalent (detected in 67% of the *Klebsiella* spp. genomes) but limited to a single farm, whereas *bla*_CTX−M−1_ was relatively widespread and detected in five farms. Unlike this study, an earlier study reported that *bla*_CTX−M−15_ is the dominant variant, followed by *bla*_CTX−M−27_ in ESBL-*E. coli* isolates from US dairy farms (Afema et al., 2018). However, *bla*_CTX−M−15_, which was detected only in one *Klebsiella* isolate in the current study, was reported as the most prevalent *bla*_CTX−M_ variant detected in *Klebsiella* isolates from humans in different parts of the US (Castanheira et al., 2014), and elsewhere (Bonnet, 2004; Calbo and Garau, 2015). In addition, *bla*_CTX−M−15_ was also reported from ESBL-*Klebsiella pneumoniae* in France (Haenni et al., 2014), ESBL-*K. ozaena* in Italy (Stefani et al., 2014) and ESBL-*K. oxytoca* in Egypt (Ahmed and Shimamoto, 2011) that were isolated from dairy cows.

In this study, *bla*_TEM_ was another frequently detected beta-lactamase gene. Most *bla*_TEM_ variants detected in this study have not been functionally characterized to determine whether ESBL or non-ESBL (Table 1). The ESBL detected in this study include *bla*_TEM −16, −29_, and _−163_. The narrow-spectrum *bla*_TEM−1B_ variant was found in 95% of ESBL-*Klebsiella* isolates carrying the *bla*_TEM_ gene, but further research is needed to determine if it provides a selective advantage to the host bacteria.

Most *Klebsiella* isolates harbored only one *bla*_TEM_ variant, except two from the same farm, which had 17 and 9 variants each on the same contig, likely due to horizontal gene transfer or accumulation of mutation due to persistent exposure to antibiotics, raising concerns about the spread of these genes via MGEs (Carattoli, 2013).

To our knowledge, no previous studies in the US reported the *bla*_TEM_ gene from *Klebsiella* spp. in dairy farms. However, a study from Pennsylvania reported *bla*TEM in *E. coli*, but the variant specification was not provided (Donaldson et al., 2006). Various *bla*_TEM_ gene was also reported from *K. pneumoniae* isolates from humans in the US (Yigit et al., 2001; Paterson et al., 2003). An extensive European review revealed that several studies documented *bla*_TEM_ variants different from those observed in the current study in *E. coli* isolates from dairy cattle (Dantas Palmeira and Ferreira, 2020). The lack of previous reports of *bla*_TEM_ and *bla*_SHV_ genes in *Klebsiella* isolates from US dairy farms likely reflects limited research rather than their actual absence, highlighting the need for ongoing surveillance of bacterial resistance in food production environments.

The rare β-lactamase genes, bla*_*OXA−1*_* and bla*_SED−1_* were detected in *K. pneumoniae*, whereas bla*_*OXY 1−7*_* was in *K. michiganensis*. To our knowledge, this is the first study to identify these resistance genes in *Klebsiella* spp. from US dairy farms. The *bla*_SED−1_ gene naturally occurs in *Citrobacter sedlakii* (Petrella et al., 2001; Naas et al., 2017). Thus, *K. pneumoniae* may have acquired this gene via horizontal gene transfer from *C. sedlakii* in the animal GIT. *K. michiganensis* is a recently described novel member of the *K. oxytoca* complex (Saha et al., 2013; Yang et al., 2022). The detection of *bla*_OXY 1−7_ variants of ESBL aligns with previous studies that reported that *K. oxytoca* inherently carries *bla*_*OXY*_, a chromosomally encoded class A beta-lactamase gene (Fournier et al., 1994). However, plasmid-associated *bla*_OXY_ was recently reported from *K. oxytoca* in Spain (Gonzalez-Lopez et al., 2009). This gene was not associated with plasmid or any other MGEs in this study, suggesting it is a chromosomal beta-lactamase gene.

In addition to beta-lactamase genes, the genomes of ESBL-*Klebsiella* isolates carry resistance genes for up to seven classes of antibiotics. This finding concurs with previous studies conducted in humans, animals, and environmental samples that showed ESBL-producing *Enterobacteriaceae* carried multiple resistance genes to structurally unrelated classes of antibiotics (Kim et al., 2005; Cantón and Coque, 2006; Tacao et al., 2014; CDC, 2019; Peirano and Pitout, 2019; Gelalcha et al., 2022; Gelalcha and Kerro Dego, 2022). Like previous studies (Kim et al., 2005; Taylor et al., 2020; Carey et al., 2022), genes mediating resistance to CIAs, such as aminoglycoside modifying genes, macrolide modifying genes, and plasmid-mediated quinolone resistance genes (PMQR), were detected in many isolates. The use of 3GC (ceftiofur), fluoroquinolones (enrofloxacin), and macrolides (erythromycin) in dairy calves might result in the co-selection and spread of these resistance genes in *Enterobacteriaceae* in dairy farms (Carey et al., 2022).

The detection of these ARGs against CIAs in many isolates is concerning, as the horizontal transfer of these genes from commensal *Klebsiella* spp. to other enteric pathogens may impede the treatment of infections caused by these pathogens (Leclercq, 2002; Poirel et al., 2012; Zhang et al., 2021). The PMQR, *qnrB*, was found in 36 (66%) isolates, making it the most frequent ARG to CIAs. This finding is consistent with previous studies that reported frequent co-occurrence of ESBL and fluoroquinolone resistance genes in *Enterobacteriaceae* (Paterson et al., 2000; Wener et al., 2010; Al-Assil et al., 2013).

The most frequently detected ARGs on the *Klebsiella* genome with the ESBL gene were the fosfomycin resistance gene (*fosA*) and a multidrug efflux pump (*oqxAB*) gene, each present in 53 (96%) of the isolates. Since its first report in 2004 in *E. coli* isolates from swine in Europe (Cantón and Coque, 2006; Gelalcha and Kerro Dego, 2022), studies have shown that the *oqxAB* gene is increasing among *Enterobacteriaceae* (Perez et al., 2013; Wong et al., 2015). According to an international report on ESBL-producing *K. pneumoniae*, the *oqxAB* gene was found in about 88% of isolates from various hospitals in different countries, including the US (Perez et al., 2013). In a separate study of *K. pneumoniae* clinical isolates from Ohio, *oqxAB* was also detected in most isolates (Perez et al., 2013). The co-occurrence of the *oqxAB* and ESBL genes has significant veterinary and human health implications. This gene is often associated with MGE and encodes a multidrug efflux pump that confers resistance to multiple classes of antibiotics, including disinfectants (Li et al., 2019).

The high prevalence of the *fosA* gene, which confers resistance to fosfomycin in *Klebsiella* spp., is worrisome. Fosfomycin is a promising broad-spectrum antibiotic used in the US to treat MDR Gram-negative organisms such as ESBL-producing *Enterobacteriaceae* and carbapenem-producing *K. pneumoniae* (Sastry and Doi, 2016). This resistance gene is considered rare in the US and was reported seven years ago from a clinical ESBL-*E. coli* from a woman patient in Pennsylvania (Alrowais et al., 2015). Thus, detecting fosfomycin resistance genes in 96% of *Klebsiella* spp. found in the GIT of dairy cattle and the farm environment is concerning because the same genes may spread to human pathogenic strains through MGEs, or the bacteria may spread to humans via the food chain or direct contact with carrier animals (Arca et al., 1997; Hou et al., 2013; Wang et al., 2017). Studies showed that *fosA* genes are relatively common in Eastern Asia, particularly China. They were detected from ESBL-*E. coli* isolates from food animals such as dairy cattle and pets (Hou et al., 2012, 2013; Wang et al., 2017). More investigation is required to determine the origin of these genes in commensal *Klebsiella* spp. since fosfomycin is not used in US dairy farms.

Apart from horizontally transferable genes, multiple chromosomal mutations that affect quinolone resistance-determining regions (QRDRs) (*gyrA*, *gyrB*, *par*C) were detected. All 55 *Klebsiella* spp. genomes had QRDRs, including *gyrA* and *par*C, the primary resistance mechanisms to (fluoro)quinolones in Gram-negative bacteria, including *Klebsiella* spp (Geetha et al., 2020).

This study found that ESBL-*Klebsiella* isolates had diverse plasmids, with an average of five replicon types per isolate. In addition, this study also identified three novel plasmid STs of the IncF family (FIA replicon types). The discovery of new plasmid STs can help understand the spread and evolution of ARGs or plasmids and control the spread of antibiotic resistance in different environments (Villa et al., 2010; Shin et al., 2012).

In this study, both narrow-host range or epidemic resistance plasmids (ERPs) of the IncF family (IncFII, IncFIA, and FIB) and broad-host-range plasmids (IncHI2A, IncHI2, IncN, and IncR) were detected. Previous studies also reported similar replicon types of plasmids in ESBL-*E. coli* isolated from dairy farms in the US (Afema et al., 2018). After the Col440I plasmid, the conjugative narrow host range, or ERPs of IncF families, were highly prevalent (seen in 89% of the *Klebsiella* genomes), with up to four subtypes of this plasmid present in each isolate. The high prevalence of ERPs in *Klebsiella* spp. is concerning as previous studies showed that these families of plasmids could easily acquire resistance genes and rapidly spread throughout *Enterobacteriaceae*, particularly among related genotypes (Carattoli, 2009; Mathers et al., 2015).

In this study, the IncFII replicon type with ST F2: A^−^: B^−^ was most frequent and was mainly associated with two co-occurring beta-lactamase genes, *bla*_CTX−M−27_ and *bla*_TEM−1B_, in over 56% (31/55) of sequenced *Klebsiella* isolates. Interestingly, these IncFII replicon type-beta-lactamase variant combinations were detected in isolates obtained from the same farm, suggesting that this specific subtype of plasmid may contribute to the spread of the two ARGs in *Klebsiella* on the farm. A previous study from the US dairy farm also reported that IncF plasmid families were associated with *bla*_CTX−M−27_ in *E. coli* (Afema et al., 2018).

The study identified the IncN plasmid ST1 linked to *bla*_CTXM−1−_ in six clonally related *K. pneumoniae* isolates from three farms and one *K. michiganensis* strain from a fourth farm. In six *Klebsiella* isolates, *bal*_CTX−M−1_ co-occurred with the *mph*(A) gene on the identical plasmids, IncN ST1. Similarly, a previous study from New York dairy farms reported the co-occurrence of *bla*_CTX−M−1_ and *mph*(A) on IncN plasmids in *Klebsiella* spp. isolated from cows with mastitis (Yang et al., 2019). The presence of the *bal*_CTX−M−1_ gene and other ARGs on identical plasmids can facilitate the emergence and persistence of MDR through co-selection (Tyson et al., 2019). In addition, unlike the present study, a previous study on US dairy cattle reported IncN plasmid in association with other *bla*_CTX−M_ genes (e.g., *bla*_CTXM−15, 27_, and _−65_) in *E. coli* (Afema et al., 2018). Another broad-host-range plasmid, IncHI2(ST3), was associated with *bla*_CTXM−65_ and/or *tet*(A) genes in five *K. pneumoniae* isolates. Previous studies also reported that the IncHI2A plasmid is associated with multiple kinds of resistance genes in *Enterobacteriaceae* isolated from animals and humans and is responsible for inter-species transmission of ARGs (Garcia Fernandez et al., 2007; Wyrsch et al., 2019).

In this study, 75% of *qnrB19* detected in *Klebsiella* genome was associated with small Col440I plasmids in all the farms, suggesting its widespread occurrence. This concurs with a recent study from Germany that reported frequent association of the *qnrB* gene with Col440I plasmids in ESBL-producing *E. coli* isolated from livestock (Juraschek et al., 2022). The wide distribution of *qnrB*-carrying Col440I plasmids among different STs of *Klebsiella* spp. and other members of *Enterobacteriaceae* suggests that they may play a crucial role in maintaining and spreading *qnrB19* genes within *Enterobacteriaceae*. However, further investigation is necessary to understand the mechanisms responsible for the Col440I plasmid spread and maintenance among *Enterobacteriaceae* even in the absence of selection pressure, as most *Klebsiella* isolates in this study were obtained from adult dairy cattle that do not use (fluoro)quinolones.

In this study, in addition to plasmids, variants of ESBL co-occurred with other ARGs such as aac(3)-Iid, aph(6)-Id, aph(3′′)-Ib, *sul2* associated with other MGEs such as Insertion sequences (IS), and Integrative Conjugative Element (ICEKp1). Previous studies widely reported the co-occurrence of ESBL and other unrelated ARGs (Lin et al., 2008; Tacao et al., 2014; Farzand et al., 2019; Astanheira and Bradford, 2021). This will facilitate the mobility of diverse ARG genes and the spread of MDR among the bacterial population. For instance, in three *Klebsiella* genomes, one or more ESBL variants co-occurred with *mph*(A) or *aac*(3)-Iid on the same contigs with IS26. This has an important implication on the epidemiology of resistance genes, as previous studies reported that IS26 helps to capture and mobilize drug resistance genes found in both chromosomes (Roy Chowdhury et al., 2015, 2018) and plasmids (Cain and Hall, 2012; Garcia et al., 2016). Studies also showed that IS26 also aids in plasmid stability and persistence by facilitating the deletion of plasmid backbone sequences whose expression is burdensome for the cell (Porse et al., 2016).

The final objective of this study was to determine the genetic relatedness and spread mechanism of ESBL-producing *Klebsiella* spp. within and between farms. This study demonstrated that the ESBL-producing *Klebsiella* spp. spreads through two modes. These include (1) clones or STs with specific *Klebsiella* spp.-plasmid-ESBL gene variant combinations were disseminated widely within and between farms, (2) plasmids harboring ESBL genes were detected in dissimilar *Klebsiella* spp. STs suggest possible horizontal transfer of the genes that might contribute to the successful spread of the bacteria in the study farms.

The extent of the two modes of ESBL transmission appeared to be different between the farms. In four farms (Farm A, B, G, and J), the spread of the ESBL genes within the farms might be primarily attributed to clonal expansion. This was evident by clusters of genetically similar isolates with the same STs-ESBL gene variant-plasmid type combinations and the same chromosomal mutations in QRDRs, indicating the clonal transmission of specific resistant *Klebsiella* clones throughout the dairy herd. For example, all *K. pneumoniae* from farms A, B, and G were identified as ST353, and they all carry *bla*_CTX−M−1_ on the plasmid, IncN (ST1). The detection of the identical ST of *K. pneumoniae* harboring the same ESBL variant on indistinguishable plasmids among dairy cattle on the same farm indicates potential animal-to-animal transmission of the bacteria or exposure to a common source (Podder et al., 2014).

The detection of the same ESBL-producing *K. pneumoniae* STs, with the same ESBL genes and plasmid profile, in three separate farms, suggests that there may be a clonal spread of the bacteria between the farms through feed, contaminated vehicles, movement of dairy cattle, and humans as described in previous studies (Davis et al., 2015; Medhanie et al., 2016; Afema et al., 2018). However, further study is needed to determine whether the spread of the bacteria is due to clonal dissemination via the mechanisms mentioned above or if there is a unique adaptation between the bacterial STs, the plasmid, and the ESBL gene.

Similarly, in Farm J, all *Klebsiella* isolates were identified as ST322 and harbored the same *bla*_SHV−2_ variants. The isolates also carried identical epidemic resistance plasmids, IncFIIK (K7: A^−^: B^−^), and novel alleles for IncFIA replicon types, suggesting the possible clonal spread of the ESBL-producing *Klebsiella* spp. in the farm. Since one of the isolates in this farm was obtained from a water sample taken from the water troughs, the animals on the farm might have independently acquired the same ST of *K. pneumoniae* by consuming the contaminated water from the same trough.

In farm M, evidence of clonal and horizontal spread of *bla*_CTX−M_ genes was observed. However, horizontal spread appeared to be the primary factor in disseminating *bla*_CTX−M_ genes on the farm. This was supported by identifying the same predominant *bla*_CTX−M_ variants, specifically the *bla*_CTX−M−27_ gene, associated with the same epidemic-resistant plasmid, IncFII (ST F2: A^−^: B^−^).

About 31 (74%) of *Klebsiella* spp. Obtained from this farm, The three novel STs, the eight distinct STs, and five Unknown STs contained *bla*_CTX−M−27_ associated with IncFII (ST F2: A^−^: B^−^) plasmids. This indicates a potential horizontal spread of this ESBL variant among diverse *Klebsiella* spp. genotypes in the farm. This concurs with the previous studies that showed narrow host range plasmids of the IncF group can acquire resistance genes and quickly spread among specific members of *Enterobacteriaceae* (Carattoli, 2009; Mathers et al., 2015). In addition, some *Klebsiella* spp. with identical STs (e.g., the novel ST7501, *n* = 6; ST469, *n* = 6; ST 687, *n* = 4; and ST239, *n* = 3) had the same *bla*_CTX−M−27_ on IncFII (ST F2: A^−^: B^−^), suggesting a possible clonal spread of these STs within the animals in the farm.

## 5 Conclusion

The study identified six families of beta-lactamase genes and 26 distinct types of horizontally transferable antibiotic resistance genes. Multiple mutations in fluoroquinolone resistance regions were detected in all isolates. Most *Klebsiella* spp. carried numerous variants of β-lactamase and other ARGs, suggesting that these bacteria are a reservoir of resistance genes. The *Klebsiella* genomes harbored 22 different plasmid replicon types, with IncFII and Col440I plasmids being the most frequently detected associated with *bla*_CTX−M_ and *qnrB19*, respectively. Most farms had genetically identical ESBL-*K. pneumoniae* strains carrying the same plasmids and ESBL genes, indicating clonal expansion and animal-to-animal transmission. Additionally, identical isolates from two farms in the same county suggest the possible inter-farm spread of the bacteria. The detection of identical ESBL variants with the same plasmid subtype in different *Klebsiella* STs suggests a possible horizontal spread of ESBL genes via plasmid transfer. These dual mechanisms of the spread of ESBL genes in *Klebsiella* spp. underscore the potential for a rapid increase in the prevalence of ESBL-*Klebsiella* spp. Given *Klebsiella*’s role as a reservoir for antibiotic resistance genes, robust strategies are needed to combat antibiotic resistance spread to reduce the potential animal and human health risks. Furthermore, the identification of novel STs of ESBL-*K. pneumoniae* and novel STs of epidemic resistance plasmids in dairy farms underscore the importance of monitoring dairy farms for emerging antibiotic-resistant pathogens.

## Data Availability

The assembled sequence data has now been submitted to the Pasteur Institute Bacterial Isolate Genome Sequence Database and can be accessed at https://bigsdb.pasteur.fr/cgi-bin/bigsdb/bigsdb.pl?db=pubmlst_klebsiella_isolates&page=submit&submission_id=BIGSdb_20240715165555_3499682_34488&view=1 BIGSdb_20240715165555_3499682_34488.

## Footnotes

1
https://sourceforge.net/projects/bbmap/

2
https://cge.food.dtu.dk/services/kmerfinder/

3
https://cge.food.dtu.dk/services/resfinder/

4
https://card.mcmaster.ca/analyze/rgi

5
https://cge.food.dtu.dk/services/plasmidfinder/

6
https://cge.food.dtu.dk/services/pmlst/

7
https://cge.food.dtu.dk/services/mobileelementfinder/

8
https://cge.food.dtu.dk/services/mlst/

9
https://bigsdb.pasteur.fr/cgi-bin/bigsdb/bigsdb.pl?db=pubmlst_klebsiella_seqdef&page=sequencequery

## Supplementary material

Supplementary Figure 1 Summary statistics of Klebsiella species genome assembly.
