# Supplementary material for: Molecular epidemiology of extended-spectrum beta-lactamase-producing-Klebsiella species in East Tennessee dairy cattle farms
Source: Front Microbiol. 2024 Sep 24;15:1439363. doi: 10.3389/fmicb.2024.1439363 (PMC11458399; doi:10.3389/fmicb.2024.1439363)
Supplement: Supplementary file 1 [file Table_1.DOCX]

Supplementary Table 1. Sample Collection and Frequency of Klebsiella spp. Isolates Across Farms in Various Counties.

| **County** | **Farm** | **Fecal Samples** | **Water** | **Feed** | **Manure** | **Total Samples Collected** | **Number of *Klebsiella* spp. Isolates Obtained from Each Farm** |
| --- | --- | --- | --- | --- | --- | --- | --- |
| 1 | A | 14 | 1 | 1 | 2 | 18 | 3 |
|  | B | 9 | 1 | 1 | 1 | 12 | 3 |
| 2 | C | 63 | 1 | 1 | 2 | 67 | 0 |
|  | D | 30 | 1 | 1 | 2 | 34 | 0 |
| 3 | E | 13 | 1 | 1 | 2 | 17 | 0 |
|  | F | 40 | 1 | 1 | 2 | 44 | 0 |
| 4 | G | 25 | 1 | **2** | 1 | 29 | 2 |
| 5 | H | 26 | 1 | 1 | 1 | 29 | 0 |
| 6 | I | 30 | 1 | 1 | 2 | 34 | 0 |
|  | L | 50 | 2 | 1 | 2 | 55 | 2 |
|  | N | 74 | 4 | 2 | 7 | 87 | 0 |
| 7 | J | 25 | 1 | 1 | 2 | 29 | 5 |
|  | K | 45 | 1 | 1 | 2 | 49 | 0 |
| 8 | M | 64 | 2 | - | 2 | 68 | 42 |
| **Total** |  | 508 | 19 | 15 | 30 | 572 | 57 |
