# Supplementary figures and images for: Molecular epidemiology of extended-spectrum beta-lactamase-producing-Klebsiella species in East Tennessee dairy cattle farms
Source: Front Microbiol. 2024 Sep 24;15:1439363. doi: 10.3389/fmicb.2024.1439363 (PMC11458399; doi:10.3389/fmicb.2024.1439363)

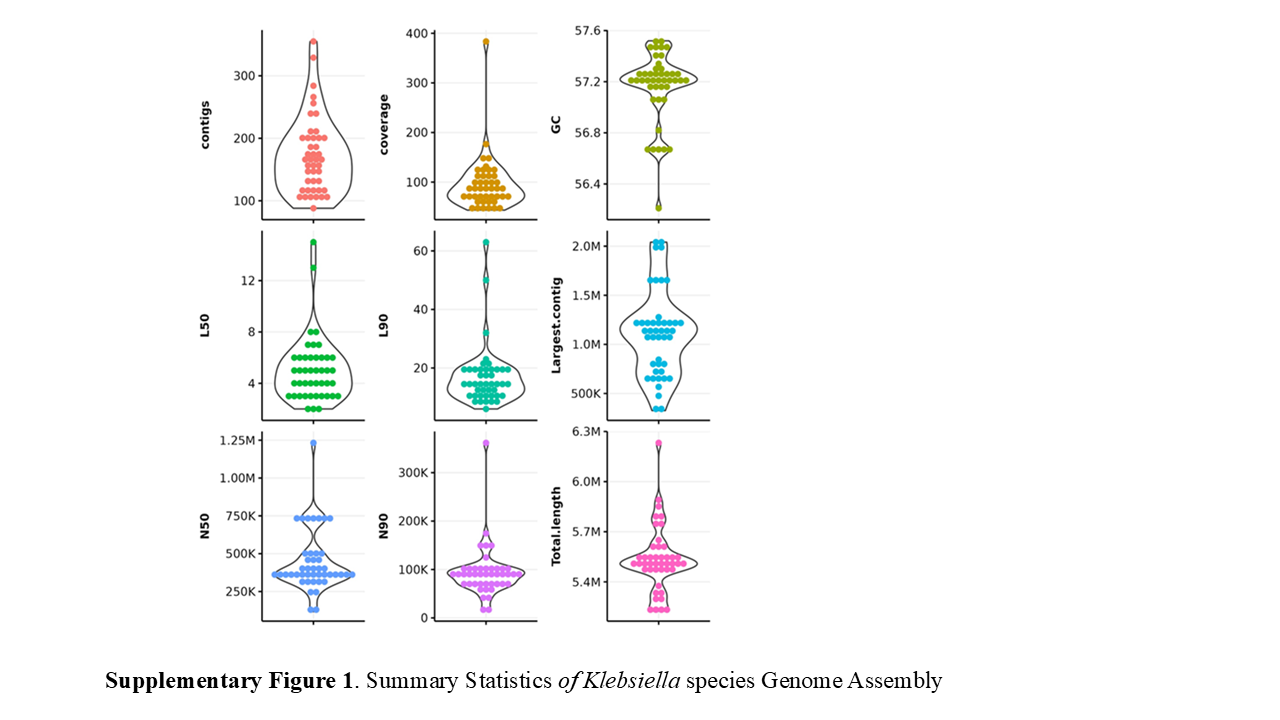

Supplement: Supplementary Figure 1 — Summary statistics of Klebsiella species genome assembly. [file Image_1.tif]
